# Outpatient screening for anxiety and depression symptoms in adolescents with type 1 diabetes - a cross-sectional survey

**DOI:** 10.1186/s13034-023-00691-y

**Published:** 2023-12-21

**Authors:** Christina Reinauer, Sascha R. Tittel, Annabel Müller-Stierlin, Harald Baumeister, Petra Warschburger, Katharina Klauser, Kirsten Minden, Doris Staab, Bettina Gohlke, Bettina Horlebein, Karl Otfried Schwab, Thomas Meißner, Reinhard W. Holl

**Affiliations:** 1 Department of General Pediatrics, Neonatology and Pediatric Cardiology, Medical Faculty, University Hospital Düsseldorf, Heinrich-Heine-University, Moorenstr. 5, 40225 Düsseldorf, Germany; 2 Institute for Epidemiology and Medical Biometry, ZIBMT, Ulm University, 89081 Ulm, Germany; 3 German Center for Diabetes Research (DZD), 85764 Munich-Neuherberg, Germany; 4 Department of Psychiatry and Psychotherapy II, Regional Hospital Günzburg, Ulm University, 89312 Günzburg, Germany; 5 Clinical Psychology and Psychotherapy, Ulm University, 89081 Ulm, Germany; 6 Department of Psychology, University of Potsdam, 14476 Potsdam, Germany; 7 Social Pediatric Center (SPZ), German Center for Pediatric and Adolescent Rheumatology, 82467 Garmisch-Partenkirchen, Germany; 8 Epidemiology Unit, German Rheumatism Research Center Berlin, a Leibniz Institute and Department of Pediatric Pneumology, Immunology and Intensive Care Medicine, Charité - Universitätsmedizin Berlin, corporate member of Freie Universität Berlin, Humboldt-Universität Berlin and Berlin Institute of Health, 10117 Berlin, Germany; 9 Department of Pediatric Pneumology, Immunology and Intensive Care Medicine, CF Center Charité - Universitätsmedizin Berlin, 13353 Berlin, Germany; 10 Pediatric Endocrinology Division, Children’s Hospital, University of Bonn, 53127 Bonn, Germany; 11 Buerger Hospital and Clementine Children Hospital, 60316 Frankfurt, Germany; 12 Faculty of Medicine, Center for Pediatrics and Adolescent Medicine, Division of Pediatric Diabetes, Medical Center, University of Freiburg, 79110 Freiburg, Germany

**Keywords:** Adolescents, Mental health, Type 1 diabetes, Anxiety, Depression

## Abstract

**Background:**

The daily demands of type 1 diabetes management may jeopardize adolescents’ mental health. We aimed to assess anxiety and depression symptoms by broad-scale, tablet-based outpatient screening in adolescents with type 1 diabetes in Germany.

**Methods:**

Adolescent patients with type 1 diabetes mellitus (n = 2,394; mean age 15.4 y [SD 2.0]; 50.7% male) were screened for anxiety (GAD-7) and depression symptoms (PHQ-9) by self-report questionnaires and linked to clinical data from the DPV patient registry. Logistic regression was used to estimate the contribution of clinical parameters to positive screening results.

**Results:**

Altogether, 30.2% showed a positive screening (score ≥ 7 in either test), and 11.3% reported suicidal ideations or self-harm. Patients with anxiety and depression symptoms were older (15.7 y [CI 15.5–15.8] vs. 15.3 y [CI 15.2–15.4]; p < 0.0001), had higher HbA1c levels (7.9% [CI 7.8-8.0] (63 mmol/mol) vs. 7.5% [CI 7.4–7.5] (58 mmol/mol); p < 0.0001), and had higher hospitalization rates. Females (adjusted odds ratio (aOR) 2.66 [CI 2.21–3.19]; p < 0.0001), patients > 15 years (aOR 1.40 [1.16–1.68]; p < 0.001), who were overweight (aOR 1.40 [CI 1.14–1.71]; p = 0.001), with HbA1c > 9% (> 75 mmol/mol; aOR 2.58 [1.83–3.64]; each p < 0.0001), with a migration background (aOR 1.46 [CI 1.17–1.81]; p < 0.001), or smoking (aOR 2.72 [CI 1.41–5.23]; p = 0.003) had a higher risk. Regular exercise was a significant protective factor (aOR 0.65 [CI 0.51–0.82]; p < 0.001). Advanced diabetes technologies did not influence screening outcomes.

**Conclusions:**

Electronic mental health screening was implemented in 42 centers in parallel, and outcomes showed an association with clinical parameters from sociodemographic, lifestyle, and diabetes-related data. It should be integrated into holistic patient counseling, enabling early recognition of mild mental health symptoms for preventive measures. Females were disproportionally adversely affected. The use of advanced diabetes technologies did not yet reduce the odds of anxiety and depression symptoms in this cross-sectional assessment.

**Supplementary Information:**

The online version contains supplementary material available at 10.1186/s13034-023-00691-y.

## Introduction

Growing up with type 1 diabetes (T1D) brings daily demands and responsibilities that, alongside the already difficult physical, psychological, and social changes of adolescence, increase the risk of anxiety and depression [1, 2]. There is a bidirectional longitudinal relationship between diabetes control and psychological problems; commonly deteriorating metabolic control during puberty [3] can lead to dissatisfaction and anxiety [4], while in turn, anxiety and depression are associated with insulin resistance and may hinder the consistent implementation of diabetes self-management [5–7]. Advanced diabetes technologies, e.g., automated insulin delivery (AID), may reduce the burden and improve treatment outcomes. However, these technologies still require management, such as constant monitoring of continuous glucose monitoring (CGM) data and rapid reactions in cases of technical alarms, carbohydrate intake, or hypoglycemia, all of which put adolescents under constant stress. Mental health in adolescents with chronic conditions has become a public health priority [8] because of its significant impact on their developmental trajectory, future mental health, long-term metabolic control, and adaptation [9, 10]. As symptoms of anxiety and depression may be nonspecific, unreported, or even kept secret by patients in a standard care setting, targeted screening methods can detect many mental health problems [11]. Although international guidelines have been demanding regular mental health screening for more than a decade [12, 13], it has not yet been implemented in most diabetes centers or incorporated into clinical reality [14]. We aimed to implement a broad-scale, tablet-based screening to assess anxiety and depression symptoms in adolescents with type 1 diabetes in Germany and identify those at risk for mental health problems.

In addition to collecting prevalence data, our focus was on identifying clinical correlates of anxiety and depression symptoms from sociodemographic and diabetes-related data, comorbidities, and lifestyle factors. We hypothesized that a longer duration of illness, beyond remission, may lead to chronic distress. Moreover, we assumed that female sex, a migration background, no use of diabetes technology, and previously diagnosed mental and somatic comorbidities would increase the risk for current anxiety and depression symptoms. Knowledge of risk factors and clinical correlates enables future target-group-specific prevention and intervention measures.

## Methods

### Subjects

**Fig. 1:**
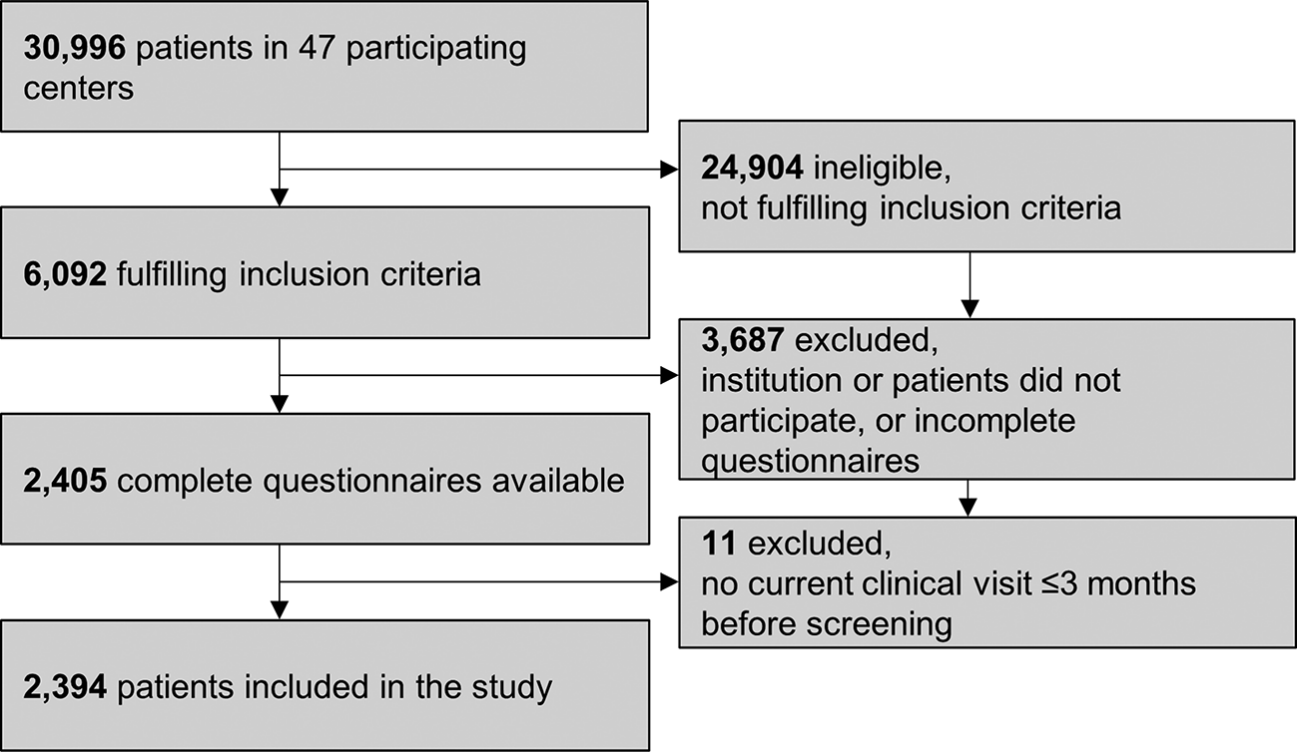
CONSORT flowchart of the study

Subjects in this cross-sectional, multicenter observational study were included within the framework of the German COACH Study (Chronic Conditions in Adolescents: Implementation and Evaluation of Patient-centered Collaborative Health Care). Adolescents with T1D, attending their regular scheduled visits at the diabetes clinics, were recruited from institutions participating in the nationwide DPV Registry (German Diabetes Prospective Follow-up Registry; see Supplementary Material 1). Inclusion criteria were age (12–21 years), diagnosis of T1D (> 10 days after manifestation), fluency in the German language to complete the questionnaires, and at least one visit to the diabetes center within the last three months. Patients with previously reported mental health problems were not excluded from this survey. Information on the sampling frame, eligible patients, and participation is given in Fig. 1. The evaluation included patients recruited between February 2019 and May 2022.

### Questionnaires

We assessed anxiety and depression symptoms during outpatient visits through two self-administered questionnaires on a tablet computer (or with paper and pencil). The GAD-7 (Generalized Anxiety Disorder Screener) is a 7-item survey measuring symptoms of generalized anxiety disorder, and the PHQ-9 (Patient Health Questionnaire) is a 9-item instrument from the Patient Health Questionnaire measuring depressive symptoms. Both questionnaires rate the frequency of symptoms on a 4-point scale (scoring 0–3) during the last two weeks, with higher scores indicating more severe symptoms and maximum scores of 21 and 27 points for the GAD-7 and PHQ-9, respectively. Both tests have high reliability and are frequently used to assess mental health problems among adolescents [15, 16]. By design, we defined a cutoff score of ≥ 7 points in either test to indicate a positive screening result [17, 18]. Results were immediately provided to the treating physician before the patient encounter so that these could be discussed at the appointment.

### Clinical data

The results were linked with clinical data that had been recorded in the DPV registry ≤ 3 months before the survey. Sociodemographic data included age, sex, body mass index (BMI), migration background, age at diabetes manifestation, and diabetes duration. Overweight was defined by BMI above the 90th percentile of the reference population [19]. Migration background was determined by the maternal country of origin – when mothers were born outside Germany, Austria, Switzerland, or Luxemburg. Diabetes-related data were HbA1c, treatment regimen (multiple daily injections [MDI], insulin pump [CSII], AID), CGM use, recent hospital admissions, and frequency of ketoacidosis or severe hypoglycemia ≤ 3 months before the survey. HbA1c values from different laboratories were mathematically adjusted to the DCCT (Diabetes Control and Complications Trial) reference range of 4.05–6.05% (20.8–42.6 mmol/mol) for comparison. Target HbA1c was defined as ≤ 7.0% (≤ 53 mmol/mol), and high HbA1c > 9.0% (> 75 mmol/mol). Analyzed comorbidities included the following diagnoses: previous or current clinical diagnosis of depression or anxiety disorder, ADHD, celiac disease, hypothyroidism, Hashimoto’s disease, and detectable thyroid autoantibodies. Due to the low incidence of schizophrenia, borderline personality disorder, and psychosis, we have combined these comorbid disorders for the analysis. Modifiable lifestyle factors included regular participation in sports and smoking.

### Statistical analysis

Descriptive statistics are presented as means, standard deviations (SDs), or numbers and percentages. Chi-square tests (for categorical variables) and Kruskal-Wallis tests (for group comparisons) were used to estimate unadjusted differences between male and female patients and those with positive or negative screening results. Two-sided p-values were adjusted for multiple testing using the Bonferroni-stepdown method. Logistic regression was used to estimate the independent contribution of each predictor on depression and anxiety. The relationship between clinical correlates and mental health is described by odds ratios (ORs) with 95% confidence intervals (CIs). Regression models were adjusted for sex, age group (≤ or > 15 years), and diabetes duration (≤ or > 6 years). All *p*-values < 0.05 were considered significant. Analyses were performed using SAS version 9.4 (build TS1M7, Cary, NC, USA), and graphical illustrations were generated using GraphPad Prism 7.0 software (GraphPad Software, San Diego, CA, USA).

## Results

### Baseline characteristics

**Fig. 2:**
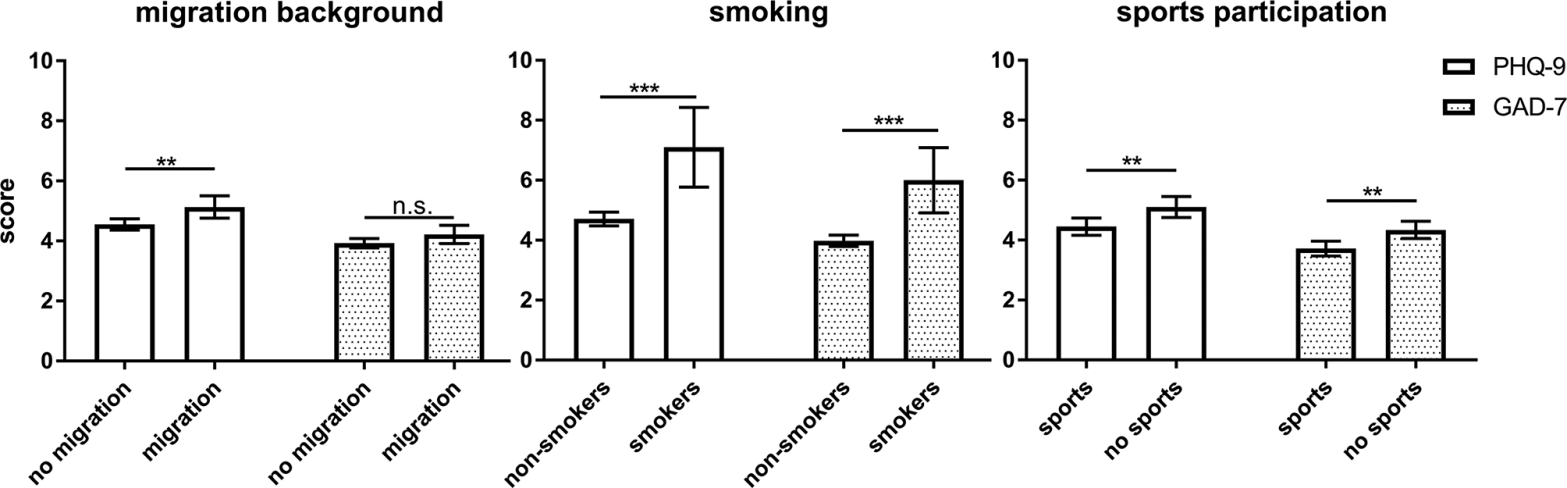
Differences in adjusted mean PHQ-9 and GAD-7 scores by migration background, smoking status, and sports participation in adolescents with T1D. Bars represent adjusted means and 95% CIs. Legend: GAD-7 = Generalized Anxiety Disorder Scale-7; PHQ-9 = Patient Health Questionnaire-9. ***p < 0.0001; **p < 0.01; *p < 0.05

A total of 2,394 adolescents with T1D (50.7% male, mean age 15.4 y [SD 2.0]) from 42 DPV centers were included in the study (Fig. 1: Flowchart). Table 1 depicts the patient characteristics of the cohort, categorized by the screening result. Altogether, 30.2% (n = 723) had a positive screening (defined as a score of ≥ 7 in either test): nearly one-fifth (19.0%, n = 454) reported symptoms of anxiety, 25.9% (n = 620) of depression, and 14.7% (n = 351) of both anxiety and depression. Severe difficulties were reported by 2.4% (n = 57): 1.7% (n = 41) scored ≥ 15 on the GAD-7, and 0.9% (n = 22) scored ≥ 20 on thePHQ-9. The ninth question in the PHQ-9, measuring suicidal ideation or self-harm (PHQ-9, item 9: “Thoughts that you would be better off dead, or of hurting yourself”), was positive in 11.3% (n = 270), with most patients (8.6%, n = 206) selecting “on several days” (score = 1) as their answer. Those with positive screening results were older (15.7 y [15.5–15.8] vs. 15.3 y [15.2–15.4]; *p* < 0.0001) and had higher HbA1c levels (7.9% [7.8-8.0] (63 mmol/mol) vs. 7.5% [7.4–7.5] (58 mmol/mol); *p* < 0.0001). In an adjusted model, a positive screening was associated with significantly higher rates of hospitalization (24.6/100 patient-years [18.4–32.9] vs. 16.0/100 patient-years [12.7–20.0]; *p* = 0.02). Table 2 contains the adjusted odds ratios (aORs) for clinical correlates of positive screening. Patients > 15.0 years, females, and those overweight or obese were more likely to report mental health problems. Those who were obese (BMI > 97th percentile) did not have an additional increased risk compared to those who were between the 90th and 97th BMI percentiles (aOR 0.86 [0.61–1.22]; *p* = 0.411). Age at diabetes manifestation was similar in patients with positive and negative screening (mean age 9.1 [SD 4.0] vs. 8.9 [4.0] years; p = 1.0). A longer diabetes duration of > 5 years led to a higher likelihood of positive screening results (adjusted for age), while a shorter course of > 1 year did not. A migration background increased the probability of a positive screening (Table 2) and was associated with higher mean screening scores (Fig. 2), but the country of origin (data available for n = 2,120) did not influence the results. Maternal countries of origin were mainly in Europe (n = 1,875; 88.4%), followed by the Middle East/Africa (n = 131; 6.2%; aOR _vs. Europe_ for positive screening 1.33 [0.91–1.95]; *p* = 0.142), Asia (n = 90, 4.3%; aOR _vs. Europe_ 1.34 [0.84–2.11]; *p* = 0.217), and others (Australia, the Americas, Canada, n = 24, 1.1%).

**Table 1:** Demographic and clinical variables stratified by screening results for anxiety and depression, unadjusted data

Variable	Total (n = 2,394)	Screening positive (n = 723)	Screening negative (n = 1,671)	*p*-value
**Sociodemographic data**				
Male sex	50.6% (n = 1,213)	34.0% (n = 246)	57.9% (n = 967)	< 0.0001
Age, years, mean (SD)	15.4 (2.0)	15.7 (2.1)	15.3 (2.0)	< 0.0001
Diabetes duration, years, mean (SD)	6.4 (4.1)	6.8 (4.1)	6.2 (4.1)	0.011
Migration background	20.6% (n = 494)	25.0% (n = 181)	18.7% (n = 313)	0.006
BMI-SDS, mean (SD) (total n = 2,316)	0.63 (1.03)	0.75 (1.01)	0.57 (1.04)	0.0004
Overweight, BMI > P90* (total n = 2,316)	26.1% (n = 605)	31.5% (n = 220)	23.8% (n = 385)	0.002
**Diabetes specific data**				
HbA1c, mean (SD) [%] [mmol/mol]	7.6 (1.4) 59.5 (14.9)	7.9 (1.5) 62.9 (16.0)	7.5 (1.3) 58.0 (14.1)	< 0.0001
MDI	46.1% (n = 1,103)	44.3% (n = 320)	46.9% (n = 783)	1.0
CSII	40.2% (n = 963)	41.1% (n = 297)	39.9% (n = 666)	1.0
AID	8.3% (n = 199)	9.1% (n = 66)	8.0% (n = 133)	1.0
CGM	80.8% (n = 1,934)	81.5% (n = 589)	80.5% (n = 1,345)	1.0
Severe hypoglycemia (≤ 3 months)	1.2% (n = 28)	1.5% (n = 11)	1.0% (n = 17)	1.0
**Comorbidities**				
Previous/current depressive disorder	3.7% (n = 89)	8.0% (n = 58)	1.9% (n = 31)	< 0.0001
Previous /current anxiety disorder	1.6% (n = 38)	2.8% (n = 20)	1.1% (n = 18)	0.026
Diagnosis of AD(H)D	3.3% (n = 80)	5.0% (n = 36)	2.6% (n = 44)	0.034
Diagnosis of schizophrenia, borderline, psychosis	2.7% (n = 65)	5.5% (n = 40)	1.5% (n = 25)	< 0.0001
Celiac disease	6.4% (n = 152)	6.5% (n = 47)	6.3% (n = 105)	1.0
Hashimoto’s thyroiditis	7.4% (n = 176)	9.4% (n = 68)	6.5% (n = 108)	0.102
Hypothyroidism	9.1% (n = 218)	9.1% (n = 66)	9.1% (n = 152)	1.0
Thyroid autoantibody (total n = 2,097)	18.4% (n = 385)	20.5% (n = 130)	17.4% (n = 255)	0.796
Self-harm or suicidal ideations (PHQ-9, item 9 ≥ 1)	11.3% (n = 270)	32.4% (n = 234)	2.2% (n = 36)	< 0.0001
**Lifestyle factors**				
Smoking (total n = 1,353)	3.0% (n = 41)	5.3% (n = 23)	2.0% (n = 18)	0.012
Sports participation (total n = 1,383)	58.4% (n = 807)	50.6% (n = 222)	62.0% (n = 585)	0.001

The total number was 2394 unless stated otherwise. Mean (SD), percentage (%), and number or total number (n). *defined by reference data from Kromeyer et al., 2001 [19]. AD(H)D = attention deficit (hyperactivity) disorder

**Table 2:** Clinical correlates of anxiety and depression symptoms, adjusted OR and 95% CI

	aOR (adjusted for age, HbA1c, and diabetes duration)	*p*
**Sociodemographic data**		
Female sex	2.66 (2.21–3.19)	< 0.0001
Migration background	1.46 (1.17–1.81)	0.0006
Age > 15 y _vs. ≤15 y_	1.40 (1.16–1.68)	0.0004
Overweight > 90. percentile _vs. ≤90 p_	1.40 (1.14–1.71)	0.0013
Diabetes duration > 1 y _vs. ≤1 y_	1.18 (0.99–1.42)	0.073
Diabetes duration > 5 y _vs. ≤5 y_	1.30 (1.08–1.57)	0.073
**Diabetes control**		
HbA1c > 7.0–9.0% _vs. ≤7.0%_ [> 53–75 mmol/mol]	1.66 (1.19–2.12)	< 0.0001
HbA1c > 9.0% _vs. ≤7.0%_ [> 75 mmol/mol]	2.58 (1.83–3.64)	< 0.0001
Severe hypoglycemia (last 3 months)	1.59 (0.72–3.50)	0.250
Ketoacidosis (last 3 months)	0.79 (0.08–7.43)	0.835
MDI	0.97 (0.81–1.18)	0.781
CSII	0.95 (0.79–1.15)	0.625
AID	1.20 (0.87–1.66)	0.257
CGM use	1.08 (0.86–1.36)	0.508
**Comorbidities**		
Previous/current clinical diagnosis of depression	3.95 (2.50–6.26)	< 0.0001
Previous/current anxiety disorder	2.20 (1.13–4.29)	0.020
Diagnosis of AD(H)D	2.81 (1.76–4.47)	< 0.0001
Diagnosis of schizophrenia/ borderline/psychosis	4.24 (2.50–7.19)	< 0.0001
Hypothyroidism	0.81 (0.59–1.11)	0.196
Hashimoto’s thyroiditis	1.19 (0.86–1.66)	0.291
Thyroid autoantibodies	1.00 (0.78–1.27)	0.980
Celiac disease	0.91 (0.63–1.31)	0.610
**Self-harm/suicidal ideations**		
PHQ-9, item9, Score = 1	18.37 (11.39–29.62)	< 0.0001
PHQ-9, item9, Score > 1	41.09 (12.05–140.14)	< 0.0001
**Lifestyle factors**		
Smoking	2.72 (1.41–5.23)	0.0027
Sports participation	0.65 (0.51–0.82)	0.0003

AD(H)D = attention deficit (hyperactivity) disorder

### Diabetes control

Patients with HbA1c above the target range also had an elevated risk of anxiety and depression symptoms (Table 2). A further subdivision into those with elevated HbA1c (> 9-10.5% [75–91 mmol/mol]) and very high HbA1c > 10.5% [> 91 mmol/mol] metabolic control showed no significant difference (aOR 0.93 [0.51–1.70]; *p* = 0.815). The use of advanced diabetes technologies, such as CGM, CSII, or AID systems, had no significant impact on the screening results in this study (Table 2). Recent severe hypoglycemia also did not increase the likelihood of positive screening. The rate of ketoacidosis did not differ between groups.

### Mental and somatic comorbidities

The youths’ current level of anxiety was associated with depressive symptoms (aOR for GAD-7 score ≥ 7 _risk for consp. PHQ−9_: 18.98 [14.65–24.58]; *p* < 0.0001) and vice versa (aOR for PHQ-9 score ≥ 7 _risk for consp. GAD−7_: 19.00 [14.67–24.60]; *p* < 0.0001). Other psychological comorbidities, such as ADHD, clinically diagnosed depression or anxiety disorders, phobias, schizophrenia, and borderline personality disorder all imposed a significant risk for a positive screening questionnaire. In contrast, somatic comorbidities, such as celiac disease, thyroid autoimmunity, or hypothyroidism, were not associated with anxiety and depression symptoms in this study (Table 2). Adjustment for antidepressant therapy (used by 1.5% (n = 35)) did not lead to statistically significant changes in the results (data not shown).

### Lifestyle factors

**Fig. 3:**
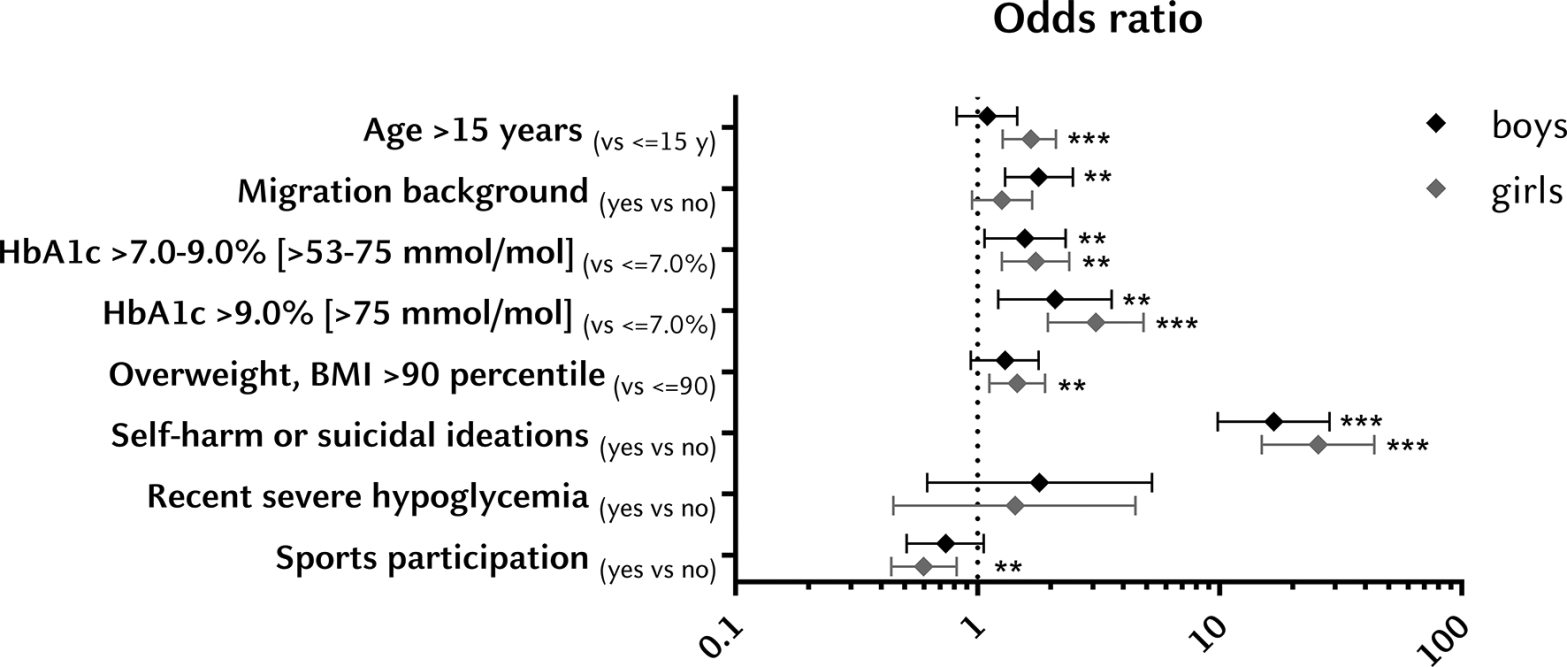
Sex-specific clinical correlates of anxiety or depression symptoms. Legend: Gray triangles represent females, black squares represent males. Bars represent ORs with 95% confidence intervals, adjusted for age, HbA1c, and diabetes duration. ***p < 0.0001; **p < 0.01; *p < 0.05

Figure 2 depicts higher mean screening scores for both questionnaires among smokers and those not engaging in regular physical exercise. The involvement in sports was associated with a lower rate of positive screening results (Table 2); however, the sex-specific analysis revealed that this was only relevant for girls (Fig. 3).

### Sex-specific analysis

Collectively, 40.4% (n = 477) of adolescent females showed a positive screening vs. 20.3% of males (n = 246; aOR 2.66 [2.21–3.19]; *p* < 0.0001), - with 35.2% of females (n = 416) screening for depression vs. 16.8% of males (n = 204; aOR 2.69 [2.21–3.26]; *p* < 0.0001), and 26.9% of females (n = 318) screening for anxiety symptoms vs. 11.2% of males (n = 136; aOR 2.91 [2.34–3.63]; *p* < 0.0001). Signs of self-harm or suicidal ideations (item 9 in the PHQ-9) were reported by 15.8% (n = 187) of females and 6.8% (n = 83) of males (aOR 2.59 [1.97–3.40]; *p* < 0.0001). The sex-specific analysis of risk factors and clinical correlates for positive screening results is depicted in Fig. 3. Sex-specific analysis revealed two subgroups (in addition to besides those with mental comorbidities) who were disproportionally affected: (i) adolescent males with a migration background (aOR 1.79 (1.30–2.48; *p* = 0.0004) and (ii) females with HbA1c levels > 9% (aOR 3.08 [1.95–4.86]; *p* < 0.0001).

## Discussion

This is the largest European adolescent T1D cohort systematically screened. Only data from the T1D Exchange in the USA include comparable – but still smaller – numbers (n = 1,714; [20], but results cannot be directly transferred to the European environment, as, e.g., payment for insulin and diabetes supplies is different in the US. We were able to demonstrate the feasibility of regular screening in the outpatient setting and that this could be implemented at 42 different centers in Germany in parallel. The primarily tablet-based screening tool with automatic evaluation and display of results for the treating physician is suitable for the young tech-savvy generation — offering a low threshold of access and could be completed independently and quickly during waiting times in the clinical offices or even from home, without significantly disrupting clinic flow [14]. Tablet-based screening may result in fewer errors due to socially desirable responses, as participants might be more candid with computers; however, more data on adolescents are needed [21]. Screening is the first step to destigmatizing mental health issues [22], but clear guidelines for advice and procedures in the event of minor abnormalities are needed. The previous reluctance regarding the implementation of screening is not only due to the hectic pace of outpatient flow and additional work involved in administering these screenings but also results from practitioners’ insecurities about the management of mildly positive results — e.g., when to refer patients to mental health care — as well as problems with making timely appointments for psychological or psychiatric support. It is an ongoing discussion whether recognizing mild cases is expedient or pathologizing normal adolescent feelings. Additionally, findings may challenge families and diabetes teams, questioning whether they are “truly” coping well with diabetes.

### Prevalence of anxiety and depression symptoms

Universal screening uncovers a high prevalence of primarily mild anxiety and depression symptoms, which seems comparable to previous meta-analyses [4–6, 20, 23–28]. Without screening, symptoms would be missed in usual clinical practice, especially in females. The rate reported by the current study (30.2%) is significantly elevated compared to, e.g., the BELLA study in the general adolescent population in Germany, where 10–15% of participants reported anxiety and depression symptoms when assessed with more extensive questionnaires (e.g., CES, DIKJ, SCARED [29]). The increased risk of self-reported symptoms was primarily found in adolescent girls. Specifically, anxiety screening results for adolescent males were similar to those reported by 11 to 17-year-old boys in the general population (11.3% in our study vs. 11.8% [29]). Of note, the reported rate of > 10% for suicidal thoughts and self-harm, twofold higher in females, is still alarming because, in diabetes, self-harm and (indirect) suicide can also be committed through insulin omission and intentional overdose, although reported suicide rates are low [30].

### Sociodemographic data and diabetes control

Demography-adjusted logistic regression revealed that being female, older than 15 years, a smoker, overweight, having a migration background, or having other comorbid mood disorders was associated with a higher likelihood of positive screening for anxiety and depression, which is comparable to previous results [6, 31]. A preliminary report on n = 1,023 adolescents by Köstner et al. (2021) had not yet shown a higher age and more inpatient admissions in the positive screening group [23]. Suboptimal HbA1c was associated with a higher likelihood of positive screening, and this association was even stronger in cases of high HbA1c levels. Consistently, various investigators have observed that mental health problems are associated with higher HbA1c [5, 26], with the notable exception of e.g., Matlock et al. (2017) [27]. We did not observe the previously described increased rate of ketoacidosis [20, 28], likely due to limiting our analysis only to incidents occurring in the 3 months prior to screening, where the rate of ketoacidosis was very low (total n = 5). Even with advanced diabetes technologies in a high-income country (> 40% used insulin pumps > 80% CGMS), anxiety and depression symptoms remained high in our cohort. Although studies are inconclusive, CGM use may have different psychological impacts [32]. We could confirm our initial hypothesis that a longer diabetes duration (> 5 years) is associated with positive screening results, which might be the consequence of long-term stress from the burden of a chronic condition, or “diabetes burnout” [33]. Adolescent females with HbA1c levels > 9% and adolescent males with a migration background need special attention to address symptoms of anxiety and depression. Youth with a migration background tend to experience worse medical care and display a higher risk of mental comorbidities, a topic that has been insufficiently studied to date [6, 34, 35].

### Comorbidities and lifestyle factors

Only mental comorbidities additionally increased the risk, but somatic comorbidities did not influence screening results, although celiac disease and thyroid disorders have been associated with depression in previous studies [36, 37]. In contrast to many unalterable risk factors, participation in regular supervised physical activity is a modifiable protective factor. In healthy youth, sports participation is negatively associated with anxiety and depression symptoms in both sexes [38, 39] and has only recently been shown to improve HbA1c levels in T1D youth [40]. Still, this is the first study demonstrating a significant association of participation in sports with mental health outcomes in T1D adolescents. Moreover, nonsmoking was associated with a lower risk of anxiety and depression symptoms, although causality remains unclear.

### Treatment regimen

Integrated care models considering psychological comorbidities offer a broader, more holistic approach to diabetes care [22]. Experts recommend specialized training for clinicians who treat adolescents with mental health problems and the application of an integrated care model for providing care in the pediatric clinic rather than exclusively referring patients to specialty mental health care [10, 22]. Physicians can start counseling adolescents themselves but require training in appropriate conversation techniques — e.g., motivational interviewing [41]. Most cases are mild and would probably be responsive to early consultation and, e.g., resource-strengthening measures [42]. Interdisciplinary management involving mental health professionals is needed in more severe cases where medication or psychotherapy is warranted [12]. Access to the “gold standard” intervention, cognitive behavioral therapy (CBT), is complicated due to long waiting times of 4–6 months for an appointment [22, 24]. Mental health apps targeting pediatric patients currently still lack quality and evaluation [43] but offer the potential to become a bridge to therapy or even to resolve mild cases in the future, as illustrated in a recent pilot trial on youthCOACH_CD_, an online depression and anxiety intervention specifically designed for adolescents with chronic diseases [44].

### Limitations

Questionnaires can only assess the dimension or a specific symptom but cannot diagnose a disorder per se. Rather, a conspicuous screening result warrants further evaluation, e.g., clinician-rated scales and examination or referral to a trained mental health expert. As the questionnaire was only offered in German, we have a 5–7% lower migration representation in study participants compared to the general German population or the DPV registry. We do not have information on all potential risk factors, such as family characteristics, socioeconomic status, level of education, diet, or other stressful life events. Due to the participants’ young age, the diabetes-associated micro- or macrovascular complication rate was too low to be analyzed. Finally, this cross-sectional analysis could not analyze the trajectories of mental health issues.

## Conclusions

Broad-scale, tablet-based outpatient screening of adolescents with type 1 diabetes in 42 centers in Germany uncovered a high prevalence (30.2%) of anxiety and depression symptoms. A holistic view is needed to cover all aspects, as clinical data from each category examined influenced screening outcomes. The use of advanced diabetes technologies did not reduce the odds of anxiety and depression symptoms in this cross-sectional assessment. While there was no association with acute metabolic derangements in the analyzed period of ≤ 3 months, the higher rate of inpatient admissions underlines the health-economic impact of mental health. While most risk factors are inherent, the avoidance of smoking and participation in sports are easily modifiable factors, mitigating the odds of psychological distress. We believe that two subgroups, adolescent girls with HbA1c levels > 9% and boys with a migration background, may also benefit from targeted future prevention campaigns.

### Electronic supplementary material

Below is the link to the electronic supplementary material.

**Supplementary Material 1**: Appendix A

## Data Availability

Data generated during this study are not publicly available because participants did not agree for their data to be shared publicly. Individual deidentified, anonymized data are available from the authors upon reasonable request (https://www.coach.klips-ulm.de/).

## Abbreviations

AID: automated insulin delivery
CGM: continuous glucose monitoring
COACH: Chronic Conditions in Adolescents: Implementation and Evaluation of Patient-centered Collaborative Health Care
CSII: continuous subcutaneous insulin infusion
GAD: Generalized Anxiety Disorder Screener
MDI: multiple daily injections
PHQ: Patient Health Questionnaire
T1D: Type 1 Diabetes
CGM: continuous glucose monitoring
