# Supplementary material for: Outpatient screening for anxiety and depression symptoms in adolescents with type 1 diabetes - a cross-sectional survey
Source: Child Adolesc Psychiatry Ment Health. 2023 Dec 21;17:142. doi: 10.1186/s13034-023-00691-y (PMC10740232; doi:10.1186/s13034-023-00691-y)
Supplement: Supplementary file 1 — Supplementary Material 1: Appendix A [file 13034_2023_691_MOESM1_ESM.docx]

***Appendix A:***

**Contributing diabetes clinics:**

Aachen - Uni-Kinderklinik RWTH, Augsburg Uni-Kinderklinik, Bad Mergentheim - Kinderdiabetologische Praxis, Berlin Virchow-Kinderklinik, Bocholt Kinderklinik, Bonn Uni-Kinderklinik, Bremerhaven Kinderklinik, Böblingen Kinderklinik, Chemnitz Kinderklinik, Darmstadt Kinderklinik Prinz. Margaret, Dresden Uni-Kinderklinik, Düsseldorf Uni-Kinderklinik, Erlangen Uni-Kinderklinik, Essen Elisabeth Kinderklinik, Frankenthal Kinderarztpraxis, Frankfurt Diabeteszentrum Rhein-Main-pädiat. Diabetologie (Clementine-Hospital), Freiburg Uni-Kinderklinik, Garmisch-Partenkirchen Kinderklinik, Göttingen Uni-Kinderklinik, Hamburg Altonaer Kinderklinik, Hamburg Kinderklinik Wilhelmstift, Hamm Kinderklinik, Hanau Kinderklinik, Heidelberg Uni-Kinderklinik, Kiel Universitäts-Kinderklinik, Konstanz Kinderklinik, München-Gauting Kinderarztzentrum, Neuss Lukas-Krankenhaus Kinderklinik, Nürnberg Cnopfsche Kinderklinik, Oldenburg Schwerpunktpraxis Pädiatrie; Passau Kinderklinik, Reutlingen Kinderklinik, Rosenheim Kinderklinik, Schweinfurt Kinderklinik, Siegen Kinderklinik, St. Augustin Kinderklinik, Stade Kinderklinik, Stolberg Kinderklinik, Traunstein Kinderklinik, Ulm Endokrinologikum Amedes, Waren-Müritz Kinderklinik, Witten Kinderarztpraxis.
